# The relationship between social determinants of health and postpartum weight retention based on the World Health Organization model: path analysis

**DOI:** 10.1186/s12889-023-15207-8

**Published:** 2023-02-14

**Authors:** Shahin Bazzazian, Giti Ozgoli, Hedyeh Riazi, Zohreh Mahmoodi, Mohammadreza Vafa, Malihe Nasiri

**Affiliations:** 1 Department of Midwifery and Reproductive Health, School of Nursing and Midwifery, Shahid Beheshti University of Medical Sciences, Tehran, Iran; 2 Midwifery and Reproductive Health Research Center, Department of Midwifery and Reproductive Health, School of Nursing and Midwifery, Shahid Beheshti University of Medical Sciences, Vali Asr Ave., Niayesh Cross Road, Niayesh Complex, 1985717443 Tehran, Iran; 3 Department of Midwifery and Reproductive Health, School of Nursing and Midwifery, Shahid Beheshti University of Medical Sciences, Tehran, Iran; 4 Social Determinants of Health Research Center, Alborz University of Medical Sciences, Karaj, Iran; 5 Nutrition Department, School of Public Health, Iran University of Medical Sciences, Tehran, Iran; 6 Department of Basic Sciences, School of Nursing and Midwifery, Shahid Beheshti University of Medical Sciences, Tehran, Iran

**Keywords:** Social Determinants of Health, Postpartum Weight Retention, Gestational weight gain, Postpartum Period

## Abstract

**Background:**

Postpartum weight retention (PPWR) causes obesity, chronic diseases, and occurring adverse maternal-fetal and neonatal outcomes. Given the social factors’ effect on health and disease and considering the lack of information on social determinants of health (SDH) effects on PPWR, this study was conducted to survey the relationship between SDH and PPWR based on the World Health Organization (WHO) model.

**Methods:**

A cross-sectional study was performed on 400 women six months after delivery in 2020. Twelve health centers were randomly selected from the three universities of Medical Sciences in the city of Tehran, Iran. Participants were selected by convenience method and based on eligibility. Questionnaires used included: Lifestyle Profile Health Promoting II, Short Form Postpartum Quality of Life Questionnaire, Multidimensional Scale of Perceived Social Support, Postpartum Social Support, Depression Anxiety Stress Scales, and questionnaires designed by reviewing the literature about breastfeeding, sleep, contraceptive, child health, unhealthy behaviors, postpartum nutritional awareness/beliefs, body satisfaction, access to postpartum care, socioeconomic status, demographic, and obstetric questionnaire. Data analysis was performed in SPSS-23, and the relationship model was examined using the path analysis method in LISREL-8.8.

**Results:**

Path analysis indicated the direct effect of six intermediate factors on PPWR including: gestational weight gain (β = 0.42), access to postpartum care (β = 0.11), postpartum nutritional awareness/beliefs (β=-0.17), anxiety (β = 0.09), sleep duration (β=-0.09), pre-pregnancy body mass index (β = 0.09). Among the structural factors, woman’s education and socioeconomic status had an indirect negative effect on PPWR. The model fit indices showed good fit (RMSE = 0/05, GFI = 0.92, CFI = 0.92, χ^2^/df = 2.17).

**Conclusion:**

The results indicate the effect of structural and intermediate determinants of health on PPWR. It is recommended to use the proposed model as an appropriate framework in the research, design, and implementation of programs to prevent and control PPWR.

## Background

Postpartum weight retention (PPWR) refers to the weight difference between pre-pregnancy and sometime after delivery [1, 2]. If this weight retention is ≤ 5 Kg, it is considered excessive [1]. In the United Kingdom, 73% [3] and in Iran, 83.5% [4] of women had not reached their pre-pregnancy weight six months after delivery. According to a systematic review and meta-analysis, two-thirds of women were more weighted six months after delivery compared to their pre-pregnancy [5]. PPWR causes obesity [6] and maternal diseases [7]. PPWR is related to several biological/behavioral factors like breastfeeding, use of hormonal contraceptives, nutrition [8], physical activity [1, 8], parity [9], pre-pregnancy BMI [3], gestational weight gain [10], smoking [11]. The association between psychological factors [12] and PPWR has been reported. Also, socioeconomic status and education [13], ethnicity [14], and age [15] were related to PPWR. Socioeconomic factors such as income, wealth, and education have been mentioned as the main reasons for many health outcomes [16]. Nowadays, social factors as the founding causes of health and disease [17] have received more global attention. Social Determinants of Health (SDH) are the conditions in which people are born, grow, live and work. These conditions are key equality factors in health [18].

Different conceptual models have been proposed to illustrate social determinant mechanisms on health [19]. In the Australian Institute of Health and Welfare (AIHW) model (2014), factors affecting health are divided into four groups: (1) the features of society and environmental factors, (2) socio-economic characteristics, awareness, attitudes, and health beliefs, (3) health behaviors and psychological and safety factors, (4) biomedical factors [20]. In the multi-level conceptual framework for the causes of childhood obesity, designed by Hawkins et al. (2018), individual factors over time are influenced by multiple upstream levels as they interact with biology and downstream levels [21]. Da Silva et al. (2013) designed a hierarchical and conceptual structure to analyze the determinants of postpartum weight variables. The first level (distal determinants) of this model, was included demographic and socio-economic factors. In the second level (intermediate determinants), variables related to the mother’s fertility and the baby are proposed. In the third level (proximal determinants), postpartum lifestyle is discussed [22]. Also, Phillips et al. (2012) presented a conceptual model and depicted the psychological factors predicting PPWR [23].

The World Health Organization’s Commission for Social Determinants of Health (CSDH) presented a model for social factors affecting health in 2010. According to this model, social factors affecting health are studied in two groups: 1- Structural social determinants and 2- Intermediate social determinants. These factors affect each other and, ultimately, health. Structural social determinants that form the social class include education, income, gender, and ethnicity (race). WHO model emphasizes the socioeconomic and political context and the structural determinants of health inequity. In this model, context, structural mechanisms, and the resultant socioeconomic situation of individuals are “structural determinants.“ The structural social determinants of health shape the health outcome through a set of intermediate determinants of health. The terms “structural determinants” and “intermediate determinants” highlight the causal priority of structural factors. Intermediate social determinants include the following categories: material conditions such as work, and access to food; behavioral and/or biological factors such as nutrition, physical activity, and genetic factors; psychosocial factors such as psychosocial stressors and social support; and the health system [24]. In this regard, PPWR can be considered a health consequence affected by structural and intermediate social factors.

According to the World Health Organization (WHO), 65.4% of Iranian women over the age of 18 were overweight or obese (BMI ≤ 25 kg/m2) in 2016 [25], and a systematic review by Djalalinia et al. (2021) showed that 71.7% of Iranian women had overweight, and 36.8% were obese [26]. Given the high prevalence of obesity and overweight in Iranian women and because PPWR is the cause of long-term obesity [27], finding factors affecting PPWR seems necessary for Iran. Some previously mentioned related factors have been investigated in the form of models such as Phillips et al. (2012) [23] and da Silva et al. (2013) [22]. Given that the social determinant of health model is one of the most completed models, and considering the WHO models, which were suggested by a group of experts in this study, are designed in a way that they can be implemented in most countries. So, the present study was conducted to investigate the relationship between SDH and PPWR based on the WHO social determinant of health 2010 model. The hypotheses of this study are: (1) Structural social determinants of health are related to PPWR (2) Intermediate social determinants of health are related to PPWR.

## Methods

A cross-sectional study was performed on 400 women six months after delivery referred to community health centers in Tehran in 2020. Four health centers were randomly selected from each of the three universities of medical sciences, including Shahid Beheshti, Tehran, and Iran (a total of 12 health centers). Participants were selected from these centers in person by convenience method and based on eligibility. The inclusion criteria were six months postpartum; not pregnant; not on weight-loss diets for six months after delivery; no chronic diseases; the last pregnancy being a singleton, alive, term, and normal; having a health record in the health center; Literate; Internet access; having a mobile phone; and desire to participate in the study. The exclusion criterion was refusing to complete the questionnaire. So, whenever the participant declined to complete the questionnaire, she was excluded from the study. The study objectives were explained to eligible women and invited them to participate in the study. Due to the Covid-19 pandemic, the data was gathered through an online questionnaire. For this purpose, the participants’ mobile phone numbers were received. Online filling out the questionnaire was explained to them, and their questions were answered in this regard. Then, the questionnaire link was sent to their phone number to fill it out at home. The height and weight of the participants were measured using a valid scale and tape available in health centers. Pre-pregnancy weight and gestational weight gain were extracted from the trustworthy health records of the participants. PPWR was calculated based on the difference between pre-pregnancy weight and six months after delivery weight. The sample size (400 subjects) was determined, given that in path analysis for each independent variable is usually considered 5–20 observations [28]. For this purpose, at first, the independent variables were determined based on the review of the literature, including Pre-pregnancy BMI/weight, Gestational weight gain, Weight at six months postpartum, Breastfeeding, Contraceptive methods, Childbirth type, Parity, Child’s health status, Sleep duration, Unhealthy behaviors, Women’s lifestyles, Postpartum nutritional awareness/beliefs, Depression, Anxiety and Stress, Social support, Body satisfaction, Socioeconomic status, Education, Job, Ethnicity, Access to postpartum health services and age of the mother. Then the final sample size was determined to be almost 400, considering 15 samples per independent variable.

**Fig. 1:**
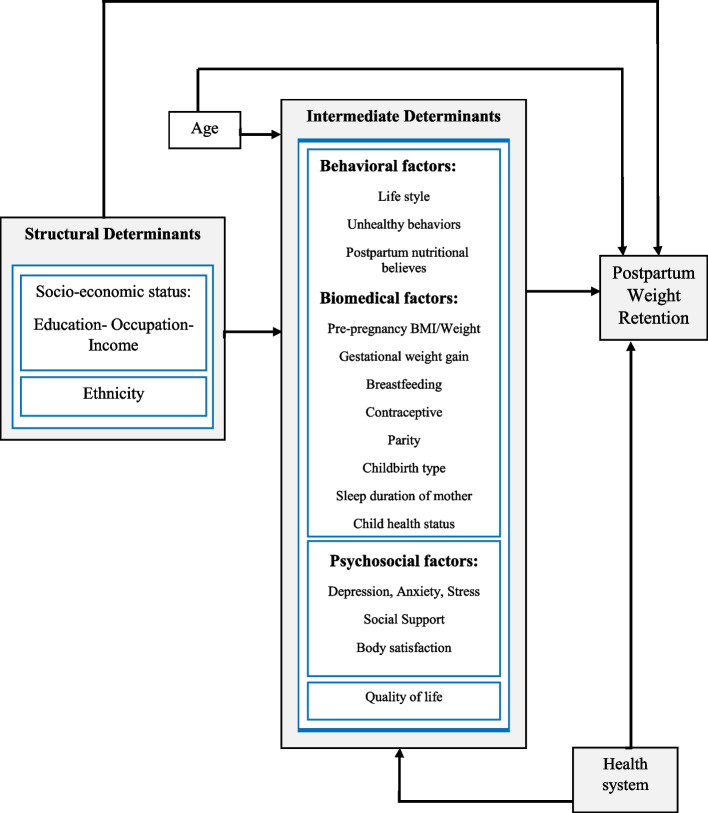
Conceptual framework of the relationship between social determinants of health and postpartum weight retention

The WHO model, which was suggested by a group of experts in this study, was studied carefully. A conceptual model of SDH affecting PPWR was designed by reviewing the literature and based on the WHO model (Fig. 1).

Data were collected using a demographic-obstetric questionnaire (including 26-item) and questionnaires assessing structural (socioeconomic status) and intermediate social determinants of PPWR (including 157-item).

Since the valid questionnaires were not for some variables, appropriate questionnaires were designed, and their validity (face and content) and reliability were evaluated. The face validity (qualitative and quantitative) of the designed questionnaires were evaluated. In the qualitative face validity, 12 postpartum mothers from the target group (six months after delivery) were asked to comment on the relevancy, level of difficulty, and ambiguity of the questionnaires items. Then proper modifications were applied to the items according to the received comments. In the quantitative face validity, each item’s impact score was determined. Items with an impact score ≥ 1.5 are considered appropriate and retained for further analysis [29]. Considering that the impact score of each item in the designed questionnaires was more than 1.5, no items were deleted.

The content validity (qualitative and quantitative) of the designed questionnaires were evaluated. In the qualitative content validity, 15 experts (Faculty members of eight universities of medical sciences) assessed the grammar, wording, and proper scoring of the designed questionnaires. Then proper modifications were applied to the items according to the received comments. In the quantitative content validity, the content validity ratio (CVR) and content validity index (CVI) were measured.

To measure CVR, 15 experts in reproductive health were asked to score the essentiality of the designed questionnaires items into three categories, including “Essential”, “Useful but not essential”, and “Not essential” from 1 to 3 respectively, based on Lawshe’s method. CVR was calculated in the following formula: CVR = (ne – (N/2)) / (N/2). In this formula, ne is the number of experts who rate an item as ‘Essential’, and N is the total number of experts. The calculated CVR was compared with the minimum acceptable CVR according to Lawshe’s table. Items with CVR more than that stated in the table for the given number of experts were considered necessary [30]. In the present study, the number of experts was 15. Based on the Lawshe table, the criteria for accepting the questions was 0.49. According to the Lawshe table, the content validity of each item in the designed questionnaires was significant, with a content validity ratio (CVR) higher than 0.49 at the *P* < 0.05 level. So, all the questions in all the designed questionnaires were retained.

To measure CVI, 15 experts in reproductive health were asked to rate the relevance of the designed questionnaires items on a four-point Likert scale from 1 to 4. CVI was calculated in the following way: Dividing the number of experts who had rated 3 or 4 for an item by the total number of experts. The Content validity index score above 0.79 is considered appropriate [31]. In the present study, based on the content validity index (CVI), all the items of the designed questionnaires had a score of 0.79 and more and are considered appropriate.

The reliability of the questionnaires was assessed by the stability and internal consistency of the questionnaires. The stability of the questionnaires was assessed by the test–retest method using the intra-class correlation coefficient (ICC). For this purpose, 20 eligible people completed the questionnaires twice with an interval of 14 days. These people were not included in the main sampling. Then, the scores obtained in the two stages were compared using the intra-class correlation coefficient (ICC). If this index is higher than 0.70, the level of stability is considered favorable [32]. This index was higher than 0.70 in all questionnaires, indicating the favorable stability of each questionnaire in the present study.

All of the present study questionnaires and their validity and reliability are as follows:

**Socioeconomic status** : Ten questions were designed by reviewing the literature and questionnaires in this field [33–36] to assess the participants’ income, economic status, parents’ education, housing, concern for financial status, and satisfaction with their car. Its validity (face and content) and reliability (using the test-retest method) were confirmed (Impact Score = 2.03–4.39; total CVR = 0.61; total CVI = 0.91–0.99; ICC = 0.82).

**Multidimensional Scale of Perceived Social Support (MSPSS) ** [37]: The psychometrics of the Persian version of this 12-item scale were assessed in Iran [38]. Its reliability was determined in the present study with Cronbach’s alpha = 0.91.

**Postpartum Social Support questionnaire of Winefield & Tiggemann** [39]: Validity and reliability of the Persian version of this 6- item questionnaire were assessed in Iran [40]. In the present study, its reliability was determined using the test-retest method with Intra-class correlation (ICC) = 0.91.

**Depression Anxiety Stress Scales (DASS-21)** [41]: The psychometric properties of the Persian version of this 21-item scale were assessed in Iran [42]. In the present study, its reliability was determined with Cronbach’s alpha 0.90 for the depression domain, α = 0.79 for the anxiety domain, and α = 0.90 for the stress domain.

**Postpartum Quality of Life (PQOL)** [36]: The psychometric properties of the short form of this questionnaire (SF-PQOL), including 13-item, were confirmed in Iran [43]. In the present study, SF-PQOL was used, and its reliability was determined using the test-retest method with ICC = 0.92.

**Lifestyle Profile Health Promoting II (HPLP II)** [44]: The validity and reliability of this 52-item questionnaire were confirmed in Iran [45]. Its reliability was determined in the present study with Cronbach’s alpha of 0.94.

**Access to health system questionnaire** : The validity and reliability of this 9-item questionnaire were assessed in Iran [46]. Its reliability was determined with Cronbach’s alpha of 0.91 in the present study.

**Access to postpartum care** : Four questions were designed, including the type of health center (private centers/ health system), received postpartum care frequency, and the use of health system cyberspace. Its validity (face and content) and reliability (using the test-retest method) were confirmed (Impact Score = 3.24–4.16; total CVR = 0.80; total CVI = 0.96–0.98; ICC = 0.83).

**Breastfeeding** : Two questions were designed to assess the type, start time, and frequency of feedings. Its validity (face and content) and reliability (using the test-retest method) were confirmed (Impact Score = 5; total CVR = 1; total CVI = 1; ICC = 0.84).

**Sleep** : Four questions were designed, including the duration of sleep, adequacy of sleep in terms of the individual, satisfaction with sleep, and having enough time to rest. Its validity (face and content) and reliability (using the test-retest method) were confirmed (Impact Score = 3.73–4.83; total CVR = 0.77; total CVI = 0.96; ICC = 0.86).

**Contraceptive** : Three questions were designed to assess the contraceptive method. Its validity (face and content) and reliability (using the test-retest method) were confirmed (Impact Score = 3.80–4.32; total CVR = 0.51; total CVI = 0.93–0.95; ICC = 0.85).

**Child health** : Four questions were designed to assess gestational week at birth time, birth weight, weight at six months, and the history of hospitalization of the child. Its validity (face and content) and reliability (using the test-retest method) were confirmed (Impact Score = 3.73–4.75; total CVR = 0.80; total CVI = 0.93- 1; ICC = 0.91).

**Unhealthy behaviors** : Five questions were designed about the frequency of smoking, drugs, hookah, alcohol, and exposure to cigarette smoke. Its validity (face and content) and reliability (using the test-retest method) were confirmed (Impact Score = 2.85-5; total CVR = 0.81; total CVI = 0.97- 1; ICC = 0.75).

**Postpartum nutritional awareness/beliefs** : Six questions were designed by reviewing the literature in this field [47–49]. Its validity (face and content) and reliability (using the test-retest method) (Impact Score = 2.32–4.09; total CVR = 0.78; total CVI = 0.92–0.99; ICC = 0.76).

**Body satisfaction** : Six questions were designed reviewing the literature and questionnaires in this field [50]. Its validity (face and content) and reliability (using the test-retest method) were confirmed (Impact Score = 2.58–4.66; total CVR = 0.77; total CVI = 0.89–0.98; ICC = 0.95).

## Procedure

The study process was as follows: First, the WHO model, which was suggested by a group of experts in this study, was studied carefully. Then, factors affecting the PPWR were extracted by reviewing the literature. These factors were categorized into structural and intermediate groups based on the WHO model, and each extracted variable was placed in its appropriate position. So, a model was designed based on the WHO model and scientific documents. Since the valid questionnaires were not for some variables, appropriate questionnaires were designed, and their validity and reliability were evaluated. Finally, data were collected through a cross-sectional study.

The data were analyzed using SPSS-23 and LISREL-8.8. Pearson correlation coefficient was determined to investigate the relationship between the studied variables. The direct and indirect effects of variables and their overall impact were investigated using the path analysis method. The fit indices were used for evaluating the model goodness of fit. The final path model was established following Wright’s rules for identifying all compound paths [51] and considering the designed conceptual framework based on the WHO model and scientific documents.

## Results

In this study, information was collected from 400 postpartum women. Table 1 shows the demographic characters of participants and their husbands. The mean ± sd age of the participants and their husbands was 32.53 ± 5.02 and 36.25 ± 5.25 years, respectively. The majority of participants (41.5%) and their husbands (31.5%) had a bachelor’s degree; 52% of them and 52.5% of their husbands were of Persian ethnicity. 72.25% of the participants were housekeepers, and 99% of their husbands were employed.

**Table 1:** Demographic characters of participants and their husbands

Variables		Wife N(%)	Husband N(%)
**Age**	≤ 25	34(8.5)	5(1.2)
	26–35	245(61.3)	171(42.8)
	36–45	119(29.7)	204(51)
	> 45	2(0.5)	20(5)
	**Total N(%)**	400(100%)	400(100%)
**Education**	Diploma and less	96(24)	126(31.5)
	Associate Degree	46(11.5)	46(11.5)
	Undergraduate	166(41.5)	126(31.5)
	Postgraduate and higher	92(23)	102(25.5)
	**Total N(%)**	400(100%)	400(100%)
**Ethnicity**	Persian	208(52)	210(52.5)
	Non-Persian	192(48)	190(47.5)
	**Total N(%)**	400(100%)	400(100%)
**Job**	Employed	111(27.75)	396(99)
	Unemployed	289(72.25)	4(1)
	**Total N(%)**	400(100%)	400(100%)

The mean ± sd parity was 1.83 ± 1.00, and 75.5% of the participants had delivered by cesarean section. The mean ± sd body mass index (BMI) pre-pregnancy was 24.78 ± 3.91 kg/m2, and the mean ± sd gestational weight gain was 13.23 ± 5.72 kg. Six months after delivery, 85.5% of the participants had not returned to their pre-pregnancy weight, and the mean ± sd PPWR was 5.28 ± 4.99 kg. Also, 50% of the participants had excessive weight retention (≥ 5 kg).

Most of the participants (77.5%) had average socio-economic status. The mean ± sd social support score was 60 ± 14.8, and the majority of the participants (85.3%) had high social support after childbirth. 56.5% of the participants had mild depression, 69% had mild anxiety, and 50.7% had mild stress. 69.5% of the participants had high body satisfaction.

The majority of the participants (69.3%) had a moderate level of lifestyle, and the majority (83.5%) did not have unhealthy behavior. The mean ± sd sleep duration of the participants was 7.23 ± 2.36 h. The majority of participants (46.5%) had exclusive breastfeeding up to 6 months after delivery. 90.5% of the participants used non-hormonal contraceptive methods.

Table 2 shows the correlation coefficients between SDH with PPWR. The results of the Pearson test showed a positive and significant correlation between PPWR with gestational weight gain, anxiety, and access to postpartum care. Also, it showed a significant negative correlation between PPWR with postpartum social support, postpartum nutritional awareness/beliefs, and sleep duration. The highest correlation was found between gestational weight gain and PPWR.

**Table 2:** Correlation of social determinants of health with postpartum weight retention

Variables	Postpartum weight retention	Gestational weight gain	Pre-pregnancy BMI	Depression	Anxiety	Stress	Postpartum nutritional awareness/beliefs	Lifestyle	Postpartum Quality of Life	Social Support	Postpartum Social Support
**Postpartum weight retention**	1										
**Gestational weight gain**	0.444^**^	1									
**Pre-pregnancy BMI**	**0.088**	**− 0.054**	**1**								
**Depression**	**0.082**	**0.055**	**0.060**	**1**							
**Anxiety**	0.107^*^	**0.046**	**0.079**	0.689^**^	**1**						
**Stress**	**0.059**	**0.057**	**0.050**	0.826^**^	0.722^**^	**1**					
**Postpartum nutritional awareness/beliefs**	− 0.195^**^	**− 0.029**	− 0.174^**^	**0.038**	**0.052**	**0.003**	**1**				
**Lifestyle**	**− 0.013**	**0.015**	**− 0.080**	− 0.539^**^	− 0.347**	− 0.494^**^	− 0.130^**^	**1**			
**Postpartum Quality of Life**	**0.018**	**− 0.005**	**0.088**	− 0.262^**^	− 0.149^**^	− 0.218^**^	**0.015**	0.282^**^	**1**		
**Social Support**	**− 0.086**	**− 0.090**	**− 0.077**	− 0.494^**^	− 0.284^**^	− 0.424^**^	**− 0.045**	0.461^**^	0.311^**^	**1**	
**Postpartum Social Support**	− 0.132^**^	**− 0.089**	− 0.122^*^	− 0.328^**^	− 0.182^**^	− 0.250^**^	**0.002**	0.255^**^	0.188^**^	0.504^**^	**1**
**Body Satisfaction**	**− 0.033**	**− 0.079**	**− 0.018**	− 0.324^**^	− 0.222^**^	− 0.298^**^	− 0.215^**^	0.411^**^	**0.051**	0.323^**^	0.197^**^
**Access to postpartum health services**	190^**^	0.178^**^	**0.043**	**− 0.088**	**− 0.001**	**− 0.092**	**0.014**	0.253^**^	**0.078**	**0.002**	**− 0.020**
**Parity**	**0.006**	**0.037**	0.155^**^	**0.055**	**0.039**	**0.023**	**− 0.029**	**0.009**	**0.034**	**− 0.029**	**− 0.070**
**Sleep duration**	− 0.123^*^	− 0.120^*^	0.169^**^	**− 0.046**	**− 0.089**	**− 0.071**	**0.054**	**− 0.035**	**0.060**	**0.060**	**0.077**
**Child hospitalization days**	**0.050**	**− 0.048**	**0.089**	**0.080**	0.111^*^	**0.093**	**− 0.055**	**− 0.030**	− 0.101^*^	**− 0.027**	**0.017**
**Breastfeeding times**	**− 0.063**	**− 0.002**	0.136^**^	**− 0.023**	**− 0.067**	**− 0.065**	**0.052**	**− 0.008**	0.269^**^	**0.003**	**0.017**
**Unhealthy behaviors**	**0.054**	**0.094**	**0.045**	**0.092**	**0.048**	**0.066**	**− 0.071**	**− 0.049**	**− 0.009**	− 0.145^**^	− 0.117^*^
**Socioeconomic status**	**− 0.060**	**0.046**	**− 0.092**	**− 0.064**	**− 0.002**	**− 0.024**	− 0.155^**^	0.202^**^	**− 0.005**	0.144^**^	0.166^**^
**Age of woman**	**− 0.008**	**− 0.027**	**0.065**	**− 0.034**	**0.046**	**− 0.003**	− 0.142^**^	0.109^*^	0.106^*^	**− 0.031**	**− 0.067**
**Education of woman**	**− 0.038**	**0.053**	− 0.171^**^	**− 0.020**	**0.023**	**0.072**	− 0.204^**^	**0.091**	− 0.187^**^	**0.053**	**0.056**

Variables	Body Satisfaction	Access to postpartum health services	Parity	Sleep duration	Child hospitalization days	Breastfeeding times	Unhealthy behaviors	Socioeconomic status	Age of woman	Education of woman
**Postpartum weight retention**										
**Gestational weight gain**										
**Pre-pregnancy BMI**										
**Depression**										
**Anxiety**										
**Stress**										
**Postpartum nutritional awareness/beliefs**										
**Lifestyle**										
**Postpartum Quality of Life**										
**Social Support**										
**Postpartum Social Support**										
**Body Satisfaction**	**1**									
**Access to postpartum health services**	**0.040**	**1**								
**Parity**	**− 0.032**	**− 0.054**	**1**							
**Sleep duration**	**− 0.052**	**− 0.022**	**− 0.032**	**1**						
**Child hospitalization days**	**− 0.063**	**− 0.066**	**0.029**	**0.076**	**1**					
**Breastfeeding times**	**0.002**	**− 0.016**	**0.044**	0.183^**^	− 0.119^*^	**1**				
**Unhealthy behaviors**	**0.000**	0.184^**^	**− 0.036**	**− 0.004**	**− 0.079**	**− 0.049**	**1**			
**Socioeconomic status**	0.130^**^	**0.054**	**− 0.072**	**− 0.028**	**− 0.053**	**− 0.053**	**− 0.020**	**1**		
**Age of woman**	0.140^**^	**− 0.022**	0.296^**^	− 0.145^**^	**− 0.019**	− 0.112^*^	**− 0.033**	**0.086**	**1**	
**Education of woman**	0.109^*^	**− 0.077**	− 0.135^**^	− 0.123^*^	**0.008**	**− 0.093**	− 0.131^**^	0.408^**^	0.234^**^	**1**

^*^Correlation is significant at 0.05 levels ^**^Correlation is significant at 0.01 levels

**Fig. 2:**
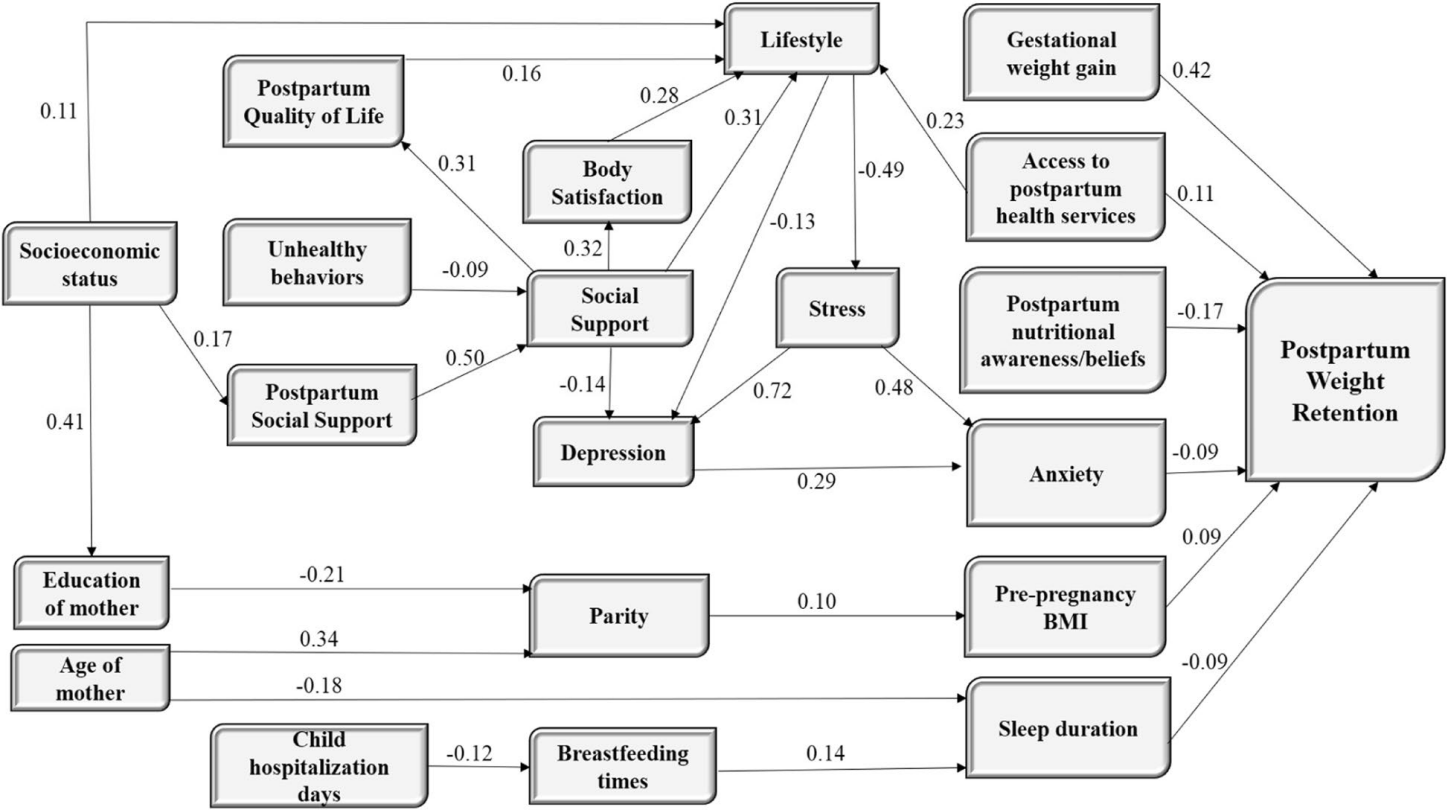
Complete path model of the effects of social determinants of health on postpartum weight retention (numbers on the lines show the standardized ß). Endogenous variable: Postpartum weight retention; Exogenous variables: Gestational weight gain, Age of mother, Access to postpartum health services, Socioeconomic status, Postpartum nutritional awareness/beliefs, Mean of Child hospitalization days, Unhealthy behaviors; Endogenous & Exogenous (Intermediate) variables: Stress, Anxiety, Depression, Lifestyle, Postpartum Quality of Life, Social Support, Postpartum Social Support, Body Satisfaction, Education of mother, Parity, Sleep duration, Pre-pregnancy BMI, Breastfeeding times

The final model of the path analysis is shown in Fig. 2. All drawn paths are significant (T-value > 1.96).

The results of path analysis indicate the direct effect of six intermediate factors, including gestational weight gain (β = 0.42), access to postpartum care (β = 0.11), postpartum nutritional awareness/beliefs (β=-0.17), anxiety (β = 0.09), sleep duration (β=-0.09), pre-pregnancy BMI (β = 0.09) were on PPWR. Gestational weight gain had the most direct positive effect, and postpartum nutritional awareness/beliefs had the most direct negative impact on PPWR. Stress had the most indirect positive effect (β = 0.06), and lifestyle had the most indirect negative effect (β = 0.03) on PPWR. Among the structural factors, woman’s education (β=-0.002) and socioeconomic status (β =-0.005) had an indirect negative effect on PPWR (Table 3).

**Table 3:** Path coefficients of social determinants affecting postpartum weight retention

Variables	Effect			Effect (standardized ß)			T- value
	Direct	Indirect	Total	Direct	Indirect	Total	
**Gestational weight gain**	0.36	-	0.36	0.42	-	0.42	9.41
**Age of mother**	-	0.02	0.02	-	0.02	0.02	-
**Stress**	-	0.06	0.06	-	0.06	0.06	-
**Access to postpartum health services**	0.30	-0.02	0.28	0.11	-0.008	0.102	2.56
**Socioeconomic status**	-	-0.01	-0.01	-	-0.005	-0.005	-
**Postpartum nutritional awareness/beliefs**	-0.29	-	-0.29	-0.17	-	-0.17	-3.91
**Mean of Child hospitalization days**	-	0.001	0.001	-	0.002	0.002	-
**Anxiety**	0.13	-	0.13	0.09	-	0.09	1.98
**Depression**	-	0.03	0.03	-	0.03	0.03	-
**Lifestyle**	-	-0.01	-0.01	-	-0.03	-0.03	-
**Postpartum Quality of Life**	-	-0.005	-0.005	-	-0.005	-0.005	-
**Social Support**	-	-0.01	-0.01	-	-0.02	-0.02	-
**Postpartum Social Support**	-	-0.04	-0.04	-	-0.008	-0.008	-
**Body Satisfaction**	-	-0.01	-0.01	-	-0.008	-0.008	-
**Education of mother**	-	-0.002	-0.002	-	-0.002	-0.002	-
**Parity**	-	0.04	0.04	-	0.009	0.009	-
**Sleep duration**	-0.88	-	-0.88	-0.09	-	-0.09	-1.99
**Pre-pregnancy BMI**	0.62	-	0.62	0.09	-	0.09	1.98
**Breastfeeding times**	-	-0.01	-0.01	-	-0.01	-0.01	-
**Unhealthy behaviors**	-	0.001	0.001	-	0.002	0.002	-

The model fit indices are shown in Table 4. Considering the relative chi-square (chi-square/degree of freedom) is less than 3, the Root Mean Square Error of Approximation (RMSEA) is less than 0.08, and the Goodness of Fit Index (GFI) and the Comparative Fit Index (CFI) are higher than 0.90, the model has a good and appropriate fit.

**Table 4:** Goodness of fit indices for the model

Index	CFI	GFI	AGFI	NFI	NNFI	IFI	RMSEA	X^2^/df
	0.92	0.92	0.89	0.86	0.89	0.92	0.055	2.17

*CFI* Comparative Fit Index, *GFI* Goodness of Fit Index, *AGFI* Adjusted Goodness of Fit Index, *NFI* Normed Fit Index, *NNFI* Non-Normed Fit Index, *IFI* Incremental Fit Index, *RMSEA* Root Mean Square Error of Approximation, *X*^2^ Chi-square test df: degree of freedom

## Discussion

This study aimed to investigate the relationship between SDH and PPWR. The path analysis in the present study showed that structural determinants, including women’s education and socioeconomic status, were indirectly related to PPWR through the effect on intermediate factors. This result is similar to the systematic review of Iranian studies, which reported a statistically significant relationship between education and PPWR [52]. The effect of women’s low education on more PPWR has also been reported by Siega-Riz et al. (2010) [53]. In the study by Zanotti et al. (2015), a higher level of education was significantly associated with maintaining a lower postpartum weight [54]. These results are similar to the results of the present study. It seems that women with higher education and higher socioeconomic status have more belief regain their pre-pregnancy weight and therefore have more control over their weight [13]. The knowledge and skills acquired through education in people with higher levels of education may affect their cognitive performance and increase their acceptance of health messages or lead to better communication and greater access to appropriate health services [24].

Six following intermediate determinants had a direct effect on PPWR:

**Gestational weight gain** had the most direct and total positive effect on PPWR. These results are similar to the results of several studies [55, 56]. Similar to the present study, a systematic review and meta-analysis showed that more weight gain in pregnancy has a significant relationship with the chance of more PPWR [10]. Gestational weight gain over Institute of Medicine (IOM) recommendations [57] has been determined as a predictor of PPWR [58]. The continuation of the pattern of activity and nutrition in pregnancy until the postpartum period [59] or biological factors such as the effect of excess gestational weight gain on increasing insulin resistance [60] may be effective in increasing PPWR [59]. This result can be helpful in health planning. In a way, health providers, in addition to recommending proper weight gain during pregnancy, should be instructed postpartum women according to gestational weight gain to control PPWR.

**Pre-pregnancy BMI** showed a direct and positive association with PPWR. In other words, with the increase in pre-pregnancy BMI, the PPWR also increases. Similarly, Celik et al. (2018) reported a significant association between pre-pregnancy BMI and PPWR [61]. On the other hand, Hollis et al. (2017) reported that low pre-pregnancy BMI was associated with increased PPWR [3]. This difference may be due to differences in pre-pregnancy weight measurement time. In a way, the time of pre-pregnancy weight measurement was 1.8 years on average before pregnancy, which is mentioned as a limitation of that research [3]. Based on path analysis in the present study, Pre-pregnancy BMI is affected by parity. Thus, with an increase in the number of pregnancies, Pre-pregnancy BMI is expected to increase, leading to more PPWR.

**Sleep duration** has a direct and negative association with PPWR. Xiao et al. (2014) systematic review study showed the impact of reduced sleep duration on the increase in PPWR [62]. Gunderson et al. (2007) reported that ≤ 5 h of sleep duration in the six months postpartum was strongly associated with ≥ 5 kg weight retention one year after delivery [63]. It seems that shorter sleep duration affects energy balance by negatively affecting glucose metabolism [64].

**Anxiety** has a direct association with PPWR. Depression and stress indirectly affected PPWR. The most indirect positive effect on PPWR belonged to stress. High stress may increase cortisol [65]. So, stress can prevent postpartum weight loss by helping to hormonal changes that increase obesity and behaviors such as overeating [66]. Phillips et al. (2014) also reported that stress in the three months after delivery had a positive and significant association with weight retention at the same time [67]. Results of a systematic review indicate a relationship between depression, anxiety, stress, and PPWR [68]. Therefore, it seems that paying attention to the mental health of postpartum women can be effective in controlling the PPWR. Faleschini et al. (2019) emphasized the protective role of social support in physical behaviors and mental health, which affects PPWR [69].

**Postpartum nutritional awareness/beliefs** showed the most direct negative effect on PPWR. In Nuss et al. (2007) study, women who maintained less than 5% of their gestational weight gain one year after delivery were more knowledgeable about nutrition than women whose gestational weight gain was equal to or more than 5% maintained [49]. It is similar to the present study and explains the necessity of planning to assess and promote the nutritional awareness of women during pregnancy and postpartum to achieve the goal of reducing PPWR. On the other hand, there is a difference between what women are advised to eat, what they feel they should eat, and what they were reported [70]. So this indicates the necessity of designing practical interventions to improve the practice of women in postpartum nutrition.

**Access to postpartum care** had both a direct positive and an indirect negative effect on PPWR. In other words, more access to the cyberspace of health systems and private centers showed an increase in PPWR. It may be due to spending too much time in cyberspace and the resulting lack of physical activity in these people leading to weight gain or preventing the expected weight loss after childbirth. Also, factors such as inadequate program content, unhealthy nutrition, or poor health behavior in private centers can justify the results of the present study. On the other hand, access to postpartum care indirectly reduces PPWR by affecting lifestyle. The total effect of this factor, which is the sum of the direct and indirect effects, is positive. So, the importance of the health system is further bolded in promoting the postpartum women’s lifestyle and reducing PPWR.

Following intermediate determinants, indirectly affected the PPWR:

**Women’s lifestyles** had the most indirect negative impact on PPWR. The effect of healthy eating and increased physical activity, as important components of lifestyle, on PPWR has been confirmed [1, 54]. In a prospective cohort study, Siega-Riz et al. (2010) identified high-calorie intake and eating behavior disorders as predictors of postpartum overweight [53]. Therefore, it seems that due to the effect of physical activity and nutrition on PPWR, with proper health planning, postpartum women can be encouraged to increase physical activity and healthy eating to reduce their overweight. According to the model of the present study, women’s lifestyle affects their stress and depression. In other words, a healthy postpartum lifestyle reduces stress and depression in women and has an indirect and negative effect on PPWR by affecting anxiety. Similarly, Van Dammen et al. (2018), in a systematic review and meta-analysis study, reported that lifestyle-related interventions in overweight/obese women of reproductive age reduce signs of depression and anxiety [71]. Lifestyle factors can affect both physical and mental health, as high mortality diseases such as cardiovascular disorders, obesity, diabetes, and cancers are affected by lifestyle [72].

**Body satisfaction** had an indirect and negative effect on PPWR. In other words, less satisfaction with the body was associated with more PPWR. Similarly, Phillips et al. (2014) reported that higher levels of body dissatisfaction at 3 and 6 months postpartum were associated with greater PPWR at nine months postpartum [67]. Hartley et al. (2017) reported in a systematic review that more weight in the postpartum period and a slow return to pre-pregnancy body size are associated with greater dissatisfaction with the body, but the mechanism of this association is still unclear [73].

**Social Support/Postpartum Social Support** had an indirect and negative effect on PPWR. According to the present model, social support affects women’s lifestyles. Some studies have reported the impact of social support on women’s lifestyle after childbirth [74, 75]. Women with children report a lack of social support as a barrier to healthy eating and physical activity [76]. The absence of a person such as a mother, friend, or other relatives of postpartum women to assist in child care, exercise, and nutrition is one of the main barriers that limit women’s ability to maintain their proper function [77].

**Unhealthy behaviors** had an indirect and positive effect on PPWR. The impact of smoking as an unhealthy behavior has also been reported in the study of Kirkegaard et al. (2015), in which women who quit smoking during pregnancy or up to 6 months after delivery were more likely to gain long-term weight than non-smokers, but this was not true for smoker women [11]. In other words, smoker women gained more weight than women who quit smoking, which this result is close to the results of the present study. In the study by Levine et al. (2012), women who continued to smoke for up to 24 weeks postpartum were more likely to be overweight than women who resumed smoking at six weeks postpartum, and this difference was statistically significant [78]. In the present study, in addition to smoking, unhealthy behaviors include several other cases (use of hookah, drugs, alcohol, exposure to secondhand smoke), while most of the existing studies are mainly about the effect of smoking (or other unhealthy behaviors) on PPWR, that may justify differences in results.

**Parity** showed an indirect and positive effect on PPWR by affecting the pre-pregnancy BMI. Similarly, in Hill et al. (2017) systematic review and meta-analysis, parity was positively related to pre-pregnancy BMI and indirectly affected the PPWR by affecting the pre-pregnancy BMI [9]. Many women with a normal BMI before their first pregnancy show an increase of more than half a unit in BMI during their second pregnancy [79]. It seems that each pregnancy leads to overweight in women, which increases cumulatively with increasing the number of pregnancies. In other words, more pregnancies are associated with higher pre-pregnancy BMI [9], and it causes an increase in PPWR.

**Breastfeeding** frequency showed an indirect negative effect on PPWR. In other words, the more frequent breastfeeding, the lower PPWR. In Krause et al. (2010) study, women with full or combination breastfeeding had less PPWR than women who had only formula-feeding six months after delivery. These researchers emphasized the negative effect of breastfeeding on PPWR [80]. In Ghobadi et al.‘s (2017) study in Iran, formula-feeding was reported as a risk factor for maintaining overweight six months after delivery [81]. It is similar to the present study. Neville et al. (2014) [82], in a systematic review, and Lambrinou et al. (2019) [83], in a review paper, reported that although some studies support the role of breastfeeding in reducing postpartum overweight, many studies are needed to achieve a reliable result.

**Child hospitalization** mean days showed an indirect and positive effect on PPWR. Kac et al. (2004) reported a significantly marginal association between child hospitalization with PPWR [84]. Siega-Riz et al. (2010) study showed a positive effect of child hospitalization on PPWR. In other words, the child hospitalization by affecting factors such as maternal sleep indirectly affected the increase in PPWR [53], similar to the present study.

**Postpartum women’s quality of life** indirectly affected the PPWR by affecting their lifestyle. The quality of life of postpartum women has been affected by social support. The effect of social support on quality of life has been confirmed in some studies [85]. Women need family support to adapt during the transition to parenthood. Social support reduces depression and acts as a link between stressors and care [85]. The Akbay & Tasci-Duran (2018) study showed that spousal support is very significant in improving the quality of life of postpartum women [86]. In the present study, the effect of quality of life on PPWR was negative. In a way, the decline in quality of life was indirectly related to the increase in postpartum overweight. Goldstein et al. (2016) also reported that worse quality of life was associated with more PPWR [12]. Improving the quality of life of postpartum women requires planning. As mentioned, postpartum women’s quality of life is affected by social support, so paying attention to increasing their social support from husbands, family, and others can improve their quality of life and ultimately cause an impact on their lifestyle and reduce PPWR.

Overall, the results of the path analysis showed that structural determinants, including women’s education and socioeconomic status, were indirectly related to PPWR through the effect on intermediate factors. Six intermediate determinants, including gestational weight gain, pre-pregnancy BMI, sleep duration, anxiety, postpartum nutritional awareness/beliefs, and access to postpartum care, directly affected the PPWR. Other determinants had an indirect effect on PPWR. Gestational weight gain had the most positive direct effect on PPWR. In other words, gestational weight gain was the highest predictor of PPWR in the study. This result, as mentioned earlier, has been reported in previous studies, and its control should be given special attention in health planning. Another important factor that had the most negative direct effect on PPWR was postpartum nutritional awareness/beliefs. Women who had more knowledge about postpartum nutrition had less PPWR, which is a valuable finding in health planning. Also, stress had the most positive indirect, and lifestyle had the most negative indirect impact on PPWR. Therefore, paying attention to stress management and improving lifestyle will be very important and effective factors in controlling PPWR.

The strength of this study is using the WHO model in SDH as a complete model that measures multiple factors. Maybe some unknown or genetic factors that are difficult/impossible to evaluate correctly were not examined, which may be a limitation of the present study. Another limitation may be recording pre-pregnancy weight from the health records. It was due to the lack of access to participants before pregnancy. Considering this recorded weight is measured according to the instructions of the Ministry of Health of Iran by health personnel, it is reliable. Another limitation, due to the numerous factors predicting PPWR, is a large number of questions and the need to spend a relatively long time to answer. So, the questionnaires with the fewest items were designed, and some short-form questionnaires were used. Also, by submitting the online questionnaire, individuals were given enough time to complete the questionnaire.

## Conclusion

The present study aimed to investigate the association between SDH and PPWR based on the WHO model. According to the results, social determinants of health (structural and intermediate) affect PPWR, confirming the hypotheses of this study. Gestational weight gain had the most positive direct effect, and postpartum nutritional awareness/beliefs had the most negative direct effects on PPWR. Also, stress had the most positive indirect, and lifestyle had the most negative indirect impact on PPWR. Therefore, to prevent or reduce PPWR, programs should be paid more attention to gestational weight gain and increase women’s awareness about postpartum nutrition’s effect on their weight. Also, counseling and interventional programs are suggested to control psychological factors such as stress/anxiety and improve the postpartum lifestyle of women. Paying attention to the impact of socioeconomic status as a structural determinant is very important, and improving it certainly requires long-term macroeconomic and political policies. Usage of the present study findings is suggested to midwives and health providers to increase women’s awareness about lifestyle, especially proper nutrition during pregnancy/postpartum, and to policymakers for long-term policies. Also, paying more attention to weight management, health, breastfeeding, and lifestyle is suggested to pregnant/postpartum women; and more social support for them is suggested to their husbands and families.

## Data Availability

The datasets used and/or analysed during the current study are available from the corresponding author on reasonable request.

## Abbreviations

PPWR: Postpartum weight retention
SDH: Social determinants of health
WHO: World Health Organization
BMI: Body mass index
CFI: Comparative Fit Index
GFI: Goodness of Fit Index
AGFI: Adjusted Goodness of Fit Index
NFI: Normed Fit Index
NNFI: Non-Normed Fit Index
IFI: Incremental Fit Index
RMSEA: Root Mean Square Error of Approximation
CVI: Content validity index
CVR: Content validity ratio
ICC: Intraclass correlation coefficient
